# A mouse dry eye model induced by topical administration of benzalkonium chloride

**Published:** 2011-01-25

**Authors:** Zhirong Lin, Xiaochen Liu, Tong Zhou, Yihui Wang, Li Bai, Hui He, Zuguo Liu

**Affiliations:** 1 State Key Laboratory of Ophthalmology, Zhongshan Ophthalmic Center, Sun Yat-sen University, Guangzhou, People’s Republic of China; 2 Eye Institute and affiliated Xiamen Eye Center of Xiamen University, Xiamen, People’s Republic of China; 3 Fujian Provincial Key Laboratory of Ophthalmology and Visual Science, Xiamen, People’s Republic of China

## Abstract

**Purpose:**

To develop a dry eye model of mouse induced by topical administration of benzalkonium chloride (BAC) and investigate the possible mechanisms.

**Methods:**

BAC at concentration of 0.2% was applied to the mouse ocular surface for 7 days. Phenol red thread tear test, tear break-up time (BUT) test, corneal inflammatory index scoring, fluorescein and rose bengal test were performed to evaluate the toxic effects of BAC on the ocular surface. Global specimens were collected on day (D) 7 and labeled with a series of antibodies including cytokeratin 10 (K10) and mucin 5AC (MUC5AC). Apoptosis of ocular surface epithelium was evaluated by in situ terminal deoxynucleotidyl transferase dUTP nick end labeling (TUNEL) assay. Histologic analysis and transmission electron microscopy (TEM) were performed on D7.

**Results:**

BAC at a concentration of 0.2% successfully induced a dry eye condition with decreased tear volume and BUTs, increased corneal fluorescein and rose bengal scores. The Inflammatory index was increased in accompanyment with higher tumor necrosis factor-α (TNF-α) expression and more inflammatory infiltration in the cornea. Immunolabeling revealed positive K10 expression in BAC-treated corneal epithelium and fewer MUC5AC-positive cells in the BAC-treated conjunctival fornix. TUNEL assay showed more apoptotic cells in the corneal basal epithelium. TEM showed that the size and intervals of the microvillis were both reduced in the corneal epithelium.

**Conclusions:**

Topical administration of 0.2% BAC in mouse induces changes resembling that of dry eye syndrome in humans, and thus, represents a novel model of dry eye.

## Introduction

Dry eye syndrome, or keratoconjunctivitis sicca (KCS), is one of the most common ocular diseases [1,2], affecting tens of million of population worldwide. With the symptoms of ocular dryness and discomfort, dry eye syndrome can lead to visual disturbance and tear film instability with potential damage to ocular surface, impacting the ability to work, read and drive at night. There is emerging evidence showing that the immunopathogenesis of dry eye is complicated and multifactoral, involving chronic inflammatory infiltration of lacrimal and salivary glands, as well as other ocular surface tissues, interruption of neuronal stimulation for tear secretion, defects of transmembrane and secretory mucin expression, as well as meibomian gland dysfunction, topical drugs preservatives, etc. Unfortunately, the precise mechanisms of dry eye syndrome were not fully understood [3].

Numerous animal models [4] have been developed to reflect the multiplicity of pathophysiological mechanisms involved in dry eye. Although the data gathered from these previous studies have provided better insights into dry eye, different models have their unique characteristics and limitations. Benzalkonium chloride (BAC) is one of the most commonly used preservative in ophthalmic solutions. The topical drugs containing preservatives have long been recognized as a potential risk of dry eye syndrome [5,6]. Recently, Xiong et al. [7] successfully developed a BAC-induced rabbit dry eye model with twice-daily topical medication, based upon the observation of reduced aqueous volume, increased fluorescein and rose bengal staining scores, and decreased goblet cell numbers.

However, our further understanding of this rabbit model was limited due to the poor availability of antibodies against rabbit proteins. In addition, it was speculated that several important pathological alterations were involved in this BAC-induced dry eye, such as inflammatory infiltration, apoptosis, and squamous metaplasia in the epithelium. The present study was therefore conducted to evaluate the effect of BAC on the ocular surface of normal mice, aiming to develop a mouse dry eye model with topical administration of BAC, and more importantly, to provide a better recognition of the preservative-induced dry eye models and their use in mechanistic and therapeutic study design.

## Methods

### Animals and procedures of benzalkonium chloride administration

Twenty male BALB/c mice (18–20 g, purchased from Shanghai SLAC laboratory animal center, Shanghai, China) were used for this study. The mice were kept in standard environment throughout the study as follows: room temperature 25 °C±1 °C, relative humidity 60%±10%, and alternating 12 h light-dark cycles (8 AM to 8 PM). All procedures were performed in accordance with the ARVO Statement for the Use of Animals in Ophthalmic and Vision Research.

The right eyes of randomly chosen 10 mice was treated with twice-daily (9 AM, 9 PM) topical administration of 5 μl of 0.2% BAC as the BAC-treated group, while the other 10 mice were treated with PBS in the right eyes as the PBS-control group. The ophthalmic preparation was adjusted to iso-osmia before used. The frequency and concentration of BAC medication were selected based on the data of our preliminary pilot experiments.

### Experimental procedure

Schirmer test, inflammatory index, fluorescein staining, break-up time of tear film (BUT), and rose bengal staining were performed in order before and during the treatment in both groups (on days 0, 1, 4, and 7). On day 7, all mice were sacrificed and the ocular global tissues were carefully dissected and harvested for histological analysis and western blot following the methods described below.

### Measurement of tear volume

The amount of tears was measured with the phenol red thread tear test using ZONE-QUICK cotton threads (Yokota, Tokyo, Japan) [8,9] on days 0, 1, 4, 7, at similar time point of the day (3 PM) in the standard environment. Animals were kept immobile by intraperitoneal injection of 1 mg pentobarbital. The lower eyelid was pulled down slightly, and a 1 mm portion of the thread was placed on the palpebral conjunctiva at a specified point approximately 1/3 of the distance from the lateral canthus of the lower eyelid. Each eye in two groups was individually tested with the eyes open for 15 s. The red portion of the thread is measured in millimeters. Each eye was tested 3×, and the average length of red portion was considered as the final length. After the test, eyes were turned closed to avoid excessive exposure and irritation of ocular surface.

### BUT, fluorescein and rose bengal staining

One microliter of 0.1% liquid sodium fluorescein was dropped into the conjunctival sac. After 3 blinks, BUTs were recorded in seconds. Ninety seconds later, corneal epithelial damage was graded with a cobalt blue filter under a slit-lamp microscope (Kanghua Science & Technology Co., Ltd, Chongqing, China). The cornea was divided into 4 quadrants, which were scored, respectively. The 4 scores were added to arrive at a final grade (total, 16 points). The fluorescein score was analyzed as previously described [10] with essential modification, briefly, as follows; absent, 0; slightly punctate staining less than 30 spots, 1; punctate staining more than 30 spots, but not diffuse, 2; severe diffuse staining but no positive plaque, 3; positve fluorescein plaque, 4.

**Figure 1:**
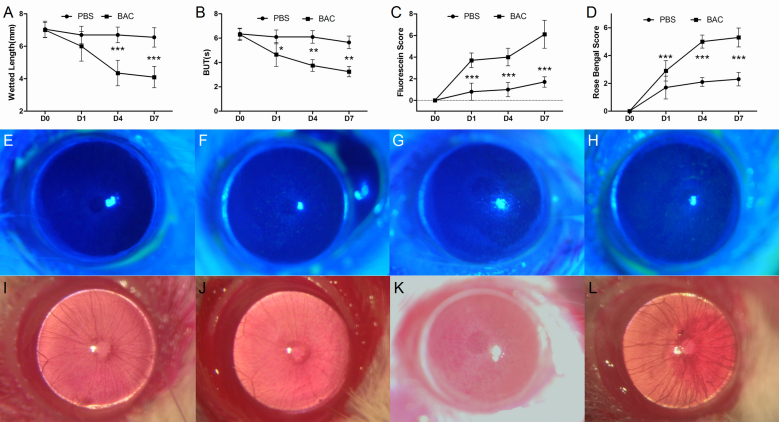
The alterations of the ocular surface after BAC treatment. Phenol red thread test showed decreased tear volume production (**A**), and decreased BUTs were described (**B**). Increased corneal fluorescein staining and rose bengal scores of ocular surface were recorded (**C** and **D**, respectively). *p<0.05, **p<0.01, ***p<0.001, respectively. Representative examples for each scale of fluorescein sodium staining (**E**-**H**, 0, 1, 2, 3 points respectively), and rose bengal staining (**I**-**L**, 0, 1, 2, 3 points respectively).

One microliter of 1% rose bengal was instilled into the conjunctival sac. Fifteen seconds later, the scores were graded under slit-lamp microscope using the Van Bijsterveld system [11]. Representative images of each scale in the grading system were provided (Figure 1E-L).

### Evaluation of inflammation

Inflammatory response was evaluated by slit lamp on days 0, 1, 4, 7. The inflammatory index was analyzed as previously described [12]. Briefly, the inflammatory index was evaluated, based on the following parameters: ciliary hyperemia (absent, 0; present but less than 1 mm, 1; present between 1 and 2 mm, 2; present more than 2 mm, 3); central corneal edema (absent, 0; present with visible iris details, 1; present without visible iris details, 2; present without visible pupil, 3); and peripheral corneal edema (absent, 0; present with visible iris details, 1; present without visible iris details, 2; present with no visible iris, 3). The final inflammatory index result was obtained by summing the scores of the different parameters divided by a factor of 9.

### In situ terminal deoxynucleotidyl transferase dUTP nick end labeling (TUNEL) labeling

To measure end-stage apoptosis, in situ TUNEL labeling was performed in frozen sections of the two groups using the DeadEnd™ Fluorometric TUNEL System (G3250; Promega, Madison, WI) according to manufacturer's instructions. Cryosections were fixed in acetone at 4 °C, rinsed with phosphate-buffer saline(PBS), permeabilized by 0.2% Triton X100, followed by incubation in equilibration buffer for 10 min. Sections were further incubated with TdT reaction mix for 60 min, then immersed in standard saline citrate to stop reaction. After a rinse, sections were counterstained with 4',6-diamidino-2-phenylindole (DAPI; Vector, Burlingame, CA), mounted, and the photo images were taken with a confocal laser scanning microscope (Fluoview FV1000; Olympus, Tokyo, Japan). For a positive control, sections were incubated in DNase I before addition of equilibration buffer, while DDW was used instead of TdT reaction mix in the negative control.

### Immunofluorescent staining

Immunofluorescent staining was performed on cryosections (6 μm thick) of the eyeballs. Sections were fixed in acetone at −20 °C, blocked, and incubated at 4 °C overnight with an anti-MUC5AC or an anti-K10 antibody (all 1:50; Santa Cruz Biotechnology, CA). After incubation in AlexaFluor488-conjugated IgG (1:1,000; Invitrogen, Carlsbad, CA), sections were counterstained with propidium iodide (Vector) or DAPI, mounted, and photographed using a confocal laser scanning microscope (Fluoview 1000; Olympus). Cornea and conjunctiva were scanned in the same area in both groups. For MUC5AC, 6 sections of each eye were used to calculate the average goblet cell number with positive cytoplasmic MUC5AC.

### Western blotting

Proteins of the cornea and conjunctiva from each group were extracted with cold RIPA buffer. Equal amounts of proteins of the cell lysates were subjected to electrophoresis on 8% SDS–PAGE and then electrophorectially transferred to PVDF membranes. After 1 h blocking in 5% BSA, the membranes were incubated with primary antibodies for TNF-α (1:400; Abcam, Cambridge, MA) and β-actin (1:10,000; Sigma, St. Louis, MO) as a loading control. After three washes with Tris-buffered saline with 0.05% Tween-20 for 10 min each, the membranes were incubated with HRP-conjugated goat anti-rabbit IgG (1:10,000; Bio-Rad, Hercules, CA) for 1 h. The specific bands were visualized by enhanced chemiluminescence reagents and recorded on film.

### Transmission electron and light microscopy

On day 7 following BAC treatment, two eyes randomly selected from each group were enucleated and fixed for 2 h in the mixture of 2.5% glutaraldehyde and 4% paraformaldehyde in PBS (pH=7.4). Then, the specimens were separated without touching the epithelium, with 2×4-mm size for both the cornea and conjunctiva. After further dissection, embedding, slicing and staining, specimens were examined and photographed with TEM (JEM2100HC; JEOL, Tokyo, Japan). Light microscopy was performed in cryosections of both groups. Tissues were stained with hematoxylin and eosin (H&E).

### Statistical analysis

Statistical analysis was performed with SPSS 16.0.0 (SPSS, Chicago, IL). One-way ANOVA was applied in all comparisons between groups. P-values less than 0.05 were considered statistically significant.

## Results

### Effects of BAC at different concentrations

The effects of BAC at different concentrations (0.1%, 0.2%, 0.25%, and 0.5%) and frequencies (2–4 times/day, daily) within 1 week were evaluated with inflammation scoring, Schirmer test, and ocular surface staining as mentioned above. Based on the preliminary results, topical instillation of BAC at 0.2% twice-daily for 7 days was considered as the optimal procedure to induce dry eye syndrome in BALB/c mice. Higher concentration of BAC caused severe damage to ocular surface, such as ulceration, large epithelial defects and neovascularization within 7 days, while BAC at 0.1% did not exhibit obvious effect regardless of the frequency and the duration (data not shown).

### Aqueous tear volume

At baseline (day 0), no significant differences were found between PBS-treated and BAC-treated groups. The tear volume declined rapidly in the BAC-treated group after the treatment, compared with the PBS-treated group. There were statistically significant differences on day 4 and 7 between the two groups (p<0.001, Figure 1A). No statistical differences were recorded at baseline.

### Stability of tear film and epithelium damage

At baseline, no differences of BUTs or scores of epithelium damage were found between the two groups. In the BAC-treated group, BUTs were significantly shortened compared with the PBS-treated group (p<0.001, Figure 1B), while scores of fluorescein sodium (Figure 1C) and rose bengal staining (Figure 1D) significantly increased after treatment (p<0.001). Within PBS-treated group, BUTs were slightly shorten without significant differences (p>0.05). However, epithelium damages also appeared from baseline in the PBS-treated group, probably due to the repeated manipulations and the toxicity of the dyes.

### Inflammation

**Figure 2:**
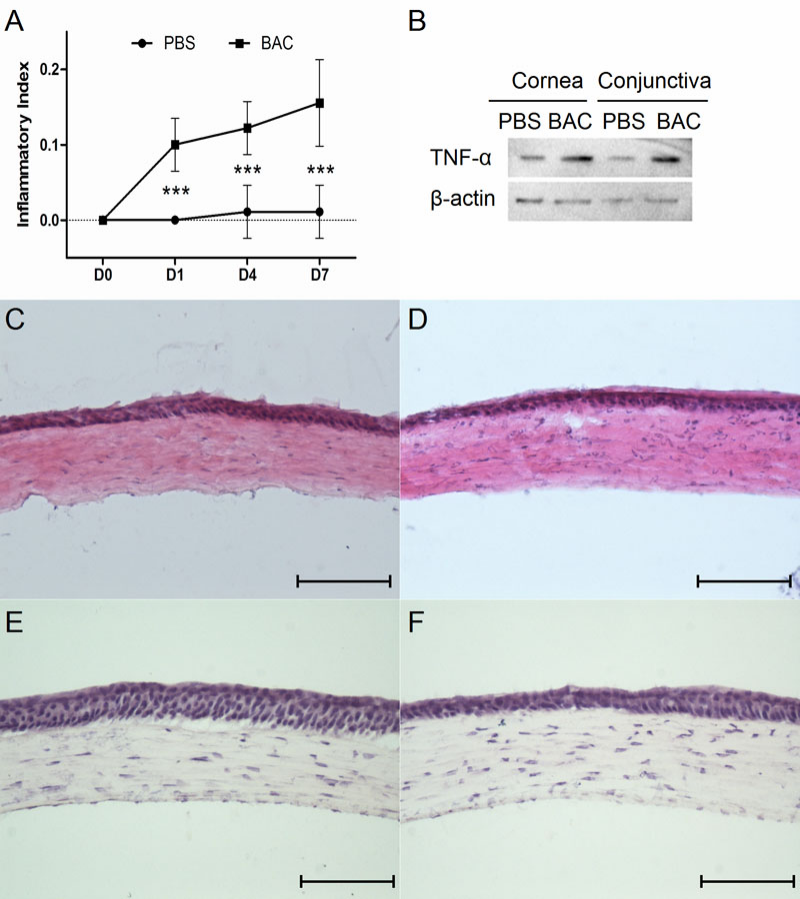
Analysis of degrees of inflammation after BAC treatment. **A**: Increased corneal inflammatory index after BAC treatment. TNF-α expression was elevated in both the cornea and conjunctiva (**B**). Representative images showed more inflammatory infiltration in peripheral corneal stroma after BAC-treatment (**D**) compared with that of the control (**C**). In central cornea, fewer layers of epithelium and more epithelial damage in BAC-treated eyes (**F**) were observed than that of the control (**E**). Scale bar=100 μm.

The corneas treated with BAC showed significant increase in inflammatory index at all of the time points (p<0.001, Figure 2A). Histological analysis of serial sections revealed that the BAC-treated group had more inflammatory cell infiltration in the peripheral cornea (Figure 2D) than the PBS-treated group (Figure 2C). In the central cornea, more epithelial damages and inflammatory infiltrations were observed in the BAC-treated eyes (Figure 2F) compared with that of the control (Figure 2E). The expression of TNF-α was upregulated in both the cornea and conjunctiva after BAC treatment (Figure 2B).

### Apoptosis and squamous metaplasia

**Figure 3:**
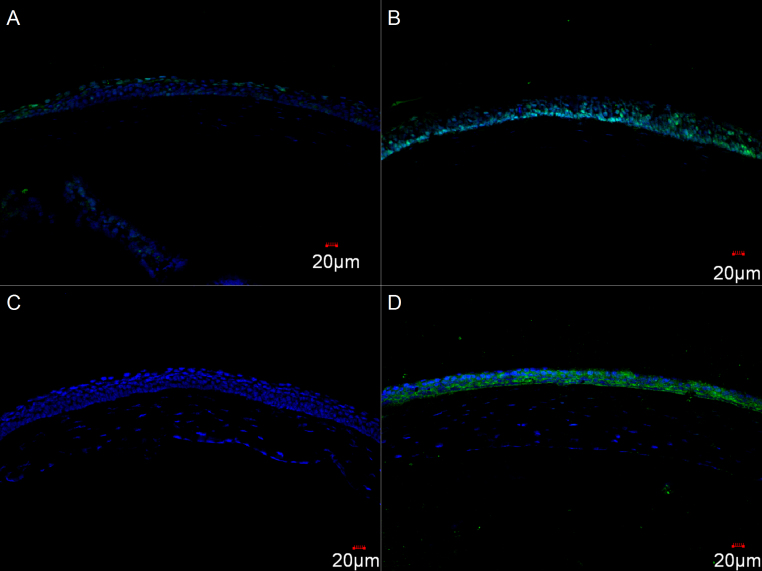
Representative images for TUNEL assay and cytokeratin 10 immunolabeling. Only a few apoptotic cells were observed in the superficial layer of corneal epithelium in control group (**A**), while much more apoptosis were recorded in corneal basal epithelium after BAC treatment (**B**). K10 was positive in corneal epithelium after BAC treatment (**D**) and negative in the control (**C**).

TUNEL assay of frozen sections revealed that BAC dramatically induced apoptosis in the corneal basal epithelium, but not in the stroma, while the PBS-treated cornea showed few apoptotic cells in the superficial corneal epithelium (Figure 3A,B), suggesting that BAC probably induced apoptosis specifically in corneal transient amplifying cells (TACs) but not terminated differentiated cells. K10 was positively expressed in the corneal epithelium after the BAC treatment for 7 days (Figure 3C,D), indicating the epithelial suqamous metaplasia.

### Density of goblet cells

**Figure 4:**
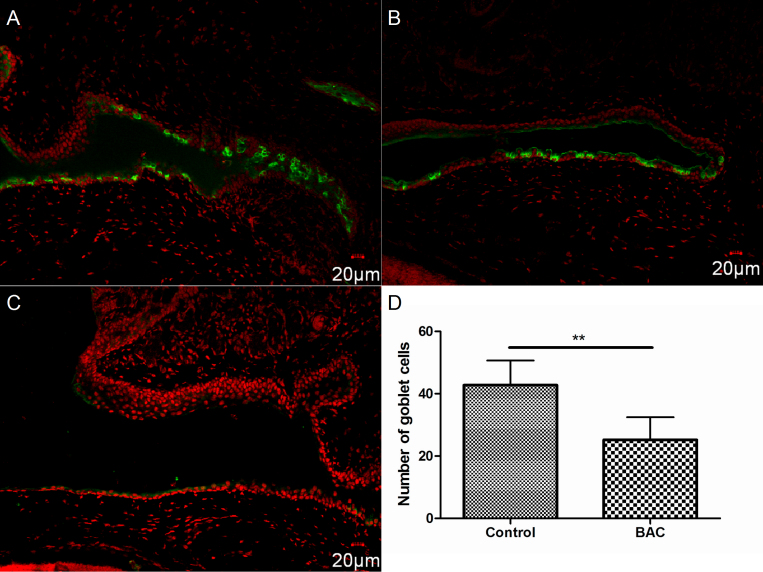
Representative images for MUC5AC staining in the conjunctiva. In the conjunctival fornix, more MUC5AC positive cells in the control group (**A**) were observed than that of the BAC-treated group (**B**), while none was recorded in the bulbar conjunctiva near limbus (**C**). The average number of MUC5AC positive cells was significantly decreased after BAC treatment (**D**). **p<0.01.

Immunofluorescent staining of MUC5AC revealed an obvious downregulation of MUC5AC-positive cells in the conjunctival fornix of the BAC group on day 7 (Figure 4D, p<0.01).

### Ultra-structural changes

**Figure 5:**
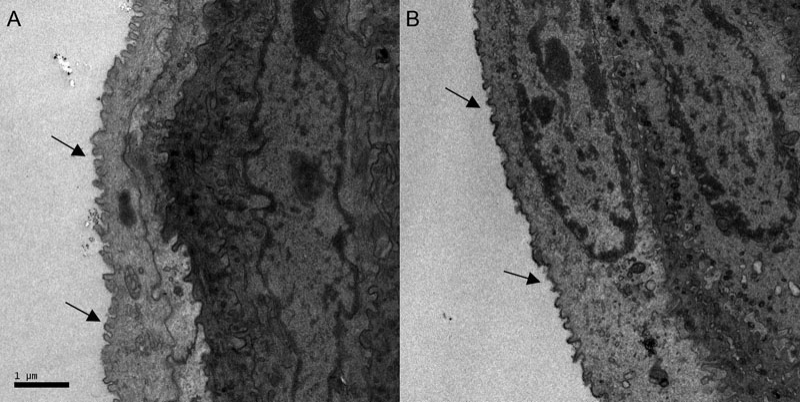
Representative images of transmission electron microscopy. Shown are the flatten microvillis with larger intervals between them on the cellular surface of epithelium after BAC treatment (**B**, black arrows). The microvillis were sharp with highdense in the control group (**A**, black arrows).

Consistent with the previous study from another group [7], the microvilli of the corneal epithelium were partially destroyed after BAC treatment. The size and intervals of microvilli were both reduced (Figure 5).

## Discussion

Dry eye has long been considered as a complicated condition which is multifactoral and can be related to any deficiencies in any of the components of the ocular surface and tear film. Developing a good animal model that simulates dry eye problems would be important for the elucidation of pathogenesis and the evaluation of promising therapeutic treatments of dry eye diseases. In this study, we introduced a mouse mode of dry eye induced by short-term topical application of a commonly used preservative, BAC, in the eye drops. The clinical assessments demonstrated that the manifestations of ocular disorders induced by topical administration of 0.2% BAC were consistent with human dry eye. Our findings revealed that BAC-induced dry eye model developed several pathological changes accordant with human dry eye syndrome, notably including inflammation, epithelial apoptosis and squamous metaplasia, which are all considered essential in the pathogenesis of dry eye syndrome.

Quaternary ammonium preservatives possess detergent properties that modify the lipid phase of the tear film and damage the integrity of epithelial cell membrane, leading to the rapid and progressive decline of tear stability. As the most commonly used preservative in ophthalmic solutions, BAC is widely known to be detrimental to ocular surface [6,10,13] and a BAC-induced rabbit model of dry eye has been recently reported [7]. Our findings further support existing data showing that BAC is able to induce a murine dry eye condition clinically similar to that in human. Squamous metaplasia, inflammatory infiltration, abnormal expression of immune mediators, and increased epithelial apoptotic rates were all observed in this BAC-induced dry eye model, and these results were consistent with those in the ocular surfaces in patients receiving prolonged topical treatment [14].

Inflammation is the key mechanism of ocular surface injury and dryness, as both the cause and consequence. Epithelial squamous metaplasia and apoptosis could be the consequences of inflammation [10,15,16]. Chronic dryness of the ocular surface resulted in gradual dysfunction of lacrimal glands and vice versa [17]. Goblet cell number could also be reduced under chronic or severe inflammation [18,19]. In our mouse model, tear secretion significantly reduced at the late phase of BAC treatment (D4 and D7). In fact, the aqueous component of tear film mainly came from the accessory lacrimal glands and conjunctiva. The tear volume reduction was probably secondary to damages and severe dryness of ocular surface, since no difference of inflammatory infiltration between two groups was observed in tissue sections of the main lacrimal glands (data not shown).

Squamous metaplasia is a hallmark of a variety of severe ocular surface disorders including chronic dry eye [20], and always happens under such as long-term deficiency of lacrimal secretion and chronic inflammatory infiltration of the ocular surface [21,22]. Our data revealed the emergence of epidermis-specific K10 expression, indicating that the non-keratinized epithelium was replaced by squamous epithelium. Li et al. [22] revealed that dryness-induced squamous metaplasia, including both abnormal epithelial differentiation and proliferation, could be observed in air-exposed limbal epithelium tissues. However, only abnormal differentiation was recorded in our mouse model, as no p63 expression was detected in the basal epithelium in the cornea of BAC-treated groups (data not shown).

The role of pathological apoptosis in the pathogenesis of dry eye has been established based on a series of studies [23-25]. Apoptotic cell death was detected on ocular tissues including cornea and especially conjunctiva, as well as the lacrimal gland. In cell cultures, preservatives could induce apoptosis at low concentrations and necrosis at high concentrations [26]. The similar phenomenon was also found in animal models and patients with dry eye [10,27]. Protection against ocular surface disorders could be achieved by suppression of apoptosis [25,28]. Our data showed notably more apoptosis in corneal basal epithelium rather than superficial epithelium after BAC treatment, while only a few apoptotic cells were found in the extremely superficial epithelium in the control. The alteration of TACs might contribute to interruption of epithelium renewal, thus leading to severe corneal epithelium damage representing high fluorescein and rose bengal staining scores. The basal epithelium presented more apoptotic cells than the superficial epithelium under BAC treatment; however, the underlying mechanisms were unclear. It was speculated that as a small molecular, BAC might easily penetrate to the basal layer containing TACs, which might be more sensitive to BAC. However, further study is needed to clarify the precise mechanisms. We did not obtain the similar results in the conjunctiva, probably due to the minor ratio of conjunctival area directly exposed to BAC drops in mouse ocular surface.

Various animal models [4,29-32] have been developed to imitate different pathophysiologic mechanisms in the development of dry eye in the past decade. However, none of them precisely mimic the clinical manifestations and the pathogenesis of human dry eye. Based on the property of preservatives, our BAC-induced dry eye models could be categorized as evaporative subtype and has its unique characteristics and limitations. Due to good availability of antibodies and transgenic variety, the mouse models are most commonly used for the clarification of immunologic mechanisms involving lacrimal gland and ocular surface inflammation. This mouse model can be appropriate especially for the study of inflammatory dry eye. As the inflammation plays an important role, the therapeutic effects of anti-inflammatory agents could be evaluated in the therapy of dry eye using this BAC-induced mouse model.
